# Construction and evaluation of risk prediction model for major adverse cardiovascular events in peritoneal dialysis patients

**DOI:** 10.3389/fneph.2026.1871318

**Published:** 2026-09-03

**Authors:** Jing Han, Xiaoli Guo, Xuejiao Liu, Jiping Sun, Fang Li, Yi Gao

**Affiliations:** 1 Department of Nephrology, Xi’an No.3 Hospital, The Affiliated Hospital of Northwest University, Xi’an, Shaanxi, China; 2 Department of Nephrology, The First Affiliated Hospital of Medical College, Xi’an Jiaotong University, Xi’an, Shaanxi, China; 3 Department of Nephrology, Shaanxi Provincial People’s Hospital, Xi’an, Shaanxi, China

**Keywords:** cardiovascular diseases, end-stage renal disease, handgrip strength, peritoneal dialysis, prediction model

## Abstract

**Background and objectives:**

This study developed and validated a robust, clinically applicable risk prediction model for cardiovascular mortality in high-risk peritoneal dialysis patients. The model enables precise individualized risk stratification, supports evidence-based clinical decisions, and facilitates targeted preventive interventions to improve long-term outcomes.

**Methods:**

A retrospective analysis was conducted on peritoneal dialysis patients at the Department of Nephrology, First Affiliated Hospital of Xi’an Jiaotong University, from June 2018 to May 2025. Cardiovascular disease served as the primary endpoint. Baseline characteristics and laboratory parameters were compared between groups. Univariate and multivariate regression analyses identified predictors of major adverse cardiovascular events. The predictive model was developed and validated using a 7:3 split after bootstrap resampling.

**Results:**

Multivariate analysis identified age, fasting blood glucose, absolute handgrip strength, and left ventricular ejection fraction as significant predictors. ROC curves (AUC > 0.6) and Kaplan–Meier curves (P < 0.05) confirmed their diagnostic and discriminatory value for major adverse cardiovascular events. A nomogram incorporating these four variables was developed. Its AUCs for predicting 3-, 5-, and 8-year cardiovascular event incidence were 0.828 (95% CI: 0.744–0.912), 0.785 (95% CI: 0.702–0.868), and 0.836 (95% CI: 0.744–0.929), respectively—indicating strong predictive discrimination. Using the optimal cut-off, patients were stratified into low- and high-risk groups. Kaplan–Meier analysis showed significant differences in cumulative event incidence at all three time points (P < 0.001), confirming good discrimination. Calibration demonstrated close agreement between predicted and observed outcomes; decision curve analysis supported robust clinical utility.

**Conclusion:**

Age, fasting blood glucose, absolute handgrip strength, and left ventricular ejection fraction are independent predictors of MACE in patients receiving PD. We have developed and internally validated a novel, interpretable nomogram-based risk prediction model that demonstrates high discriminative accuracy, calibration fidelity, and clinical utility. This tool holds substantial promise for early identification of high-risk individuals at PD initiation, thereby facilitating timely, personalized cardiovascular risk mitigation strategies.

## Introduction

Chronic kidney disease (CKD) is a progressive and irreversible clinical syndrome characterized by persistent abnormalities in kidney structure and/or function, which may ultimately progress to end-stage renal disease (ESRD). The principal therapeutic modalities for ESRD are kidney transplantation and renal replacement therapy—including hemodialysis (HD) and peritoneal dialysis(PD)—with the latter two representing the most widely implemented dialysis modalities worldwide. PD offers several clinical advantages, including better preservation of residual renal function, procedural simplicity, minimal hemodynamic instability, fewer dietary restrictions, and a lower risk of bloodstream infection compared with HD (1). However, its long-term use is associated with important limitations, such as recurrent peritonitis, inadequate small- and middle-molecule solute clearance, progressive peritoneal membrane dysfunction, and glucose-induced metabolic complications. Prolonged PD exposure predisposes patients to serious complications—including peritonitis, encapsulating peritoneal sclerosis, and MACE—which collectively contribute to substantially reduced survival and health-related quality of life relative to age-matched healthy populations. Cardiovascular disease (CVD) represents the most prevalent and life-threatening complication among PD patients and is the leading cause of mortality in this population (2). Epidemiological data indicate that the global prevalence of CVD is approximately 76.5% among HD and 65% among PD (3); moreover, CVD significantly elevates both all-cause mortality and hospitalization rates in individuals undergoing PD (4). Consequently, effective cardiovascular risk prevention and management constitute a critical priority in the care of patients receiving long-term dialysis.

Current literature predominantly employs univariate approaches to estimate CVD risk in patients undergoing PD, limiting clinical utility due to the multifactorial and interdependent nature of CVD pathogenesis in CKD. The complexity of CKD-related mortality—driven by intertwined cardiovascular, metabolic, inflammatory, and uremic mechanisms—has hindered the development of robust, multivariable prediction models for both CVD events and all-cause mortality in this population. Consequently, there remains a critical unmet need for a simple, validated, and clinically actionable risk stratification tool tailored specifically to PD patients. To address this gap, we conducted this retrospective cohort study to identify incident MACE, rigorously evaluated the independent prognostic value of baseline clinical, biochemical, and functional parameters, and developed a quantitative, multivariable nomogram grounded in statistically validated predictors. This tool enables comprehensive, individualized cardiovascular risk assessment and facilitates early identification of high-risk PD patients for targeted preventive strategies.

## Materials and methods

### Data source and study population

This study was conducted at the Peritoneal Dialysis Center, Department of Nephrology, the First Affiliated Hospital of Xi’an Jiaotong University. Patients who underwent new catheterization and regular PD at this center from June 2018 to May 2025 were included, with the dialysis mode being continuous ambulatory PD.

### Inclusion and exclusion criteria

Inclusion Criteria: Patients aged ≥18 years with PD, with dialysis duration > 3 months, no peritonitis or cardiac diseases at PD initiation, and no severe infections or other diseases affecting nutritional data and survival time.

Exclusion Criteria: Patients with indications for emergency dialysis at initiation, life expectancy < follow-up duration, recent cardio-cerebrovascular events, history of kidney transplantation or HD, or poor treatment compliance.

### Data collection and variables

Comprehensive baseline data were consecutively documented in electronic medical records at the time of PD initiation. These included: (i) demographic and clinical characteristics—namely, sex, age, height, weight, seated brachial blood pressure, primary renal diagnosis, and presence or absence of peripheral edema; (ii) standardized laboratory parameters—comprising complete blood count, serum biochemistry, spot urine analysis and peritoneal dialysate analysis; and (iii) echocardiographic indices. Peritoneal membrane transport function was assessed using a standardized 4-hour peritoneal equilibration test (PET): 2 L of 4.25% dextrose-containing dialysate was instilled and retained intraperitoneally; dialysate samples were collected at 0, 1, and 4 hours, and a concurrent venous blood sample was drawn at 1 hour. The dialysate-to-plasma sodium ratio at 1 hour served as a surrogate marker of transcellular water transport mediated by aquaporin-1 channels; the dialysate-to-plasma creatinine ratio at 4 hours (D/P Cr_4h_) was used to classify peritoneal solute transport status according to established PET criteria. Handgrip strength was measured bilaterally three times using a calibrated hydraulic dynamometer with subjects seated, elbows flexed to 90°, and feet flat on the floor; the highest value from each hand was recorded. Absolute handgrip strength was defined as the sum of the dominant and non-dominant hand values (in kilograms), and relative handgrip strength was calculated as absolute handgrip strength divided by body mass index (BMI, kg/m²).

### Endpoint events

The primary endpoint was major adverse cardiovascular events (MACE), defined as a composite event including sudden cardiac death, arrhythmia, heart failure, angina pectoris, coronary artery disease, acute coronary syndrome, peripheral vascular embolism, or hospitalization caused by cardiovascular diseases. All MACE during follow-up were independently reviewed and adjudicated by two blinded cardiologists uninvolved in enrollment or data collection; discrepancies were resolved by a third senior cardiovascular specialist.

### Statistical analysis

Normality of continuous variables was assessed using the Kolmogorov–Smirnov test with Lilliefors correction. Data conforming to a normal distribution are presented as mean ± standard deviation; non-normally distributed variables are reported as median (interquartile range, IQR). Categorical variables are expressed as frequency (percentage). Between-group comparisons were conducted using the independent samples t-test for normally distributed continuous variables, the Mann–Whitney U test for non-normally distributed continuous variables, and the chi-square test (or Fisher’s exact test where expected cell counts <5) for categorical variables. All statistical analyses were performed using SPSS Statistics version 26.0 (IBM Corp., Armonk, NY, USA).

This study preselected candidate variables based on prior research on cardiovascular risk in peritoneal dialysis patients, covering demographics, comorbidities, lab biomarkers, physical and nutritional indicators, echocardiographic parameters, and dialysis-related factors. For missing data: variables with <5% missingness were analyzed via complete-case analysis; those with >40% missingness or non-random patterns (e.g., E/e′, left atrial volume index)—which risk selection bias—were excluded; multiple imputation was avoided due to the association between missingness and underlying patient conditions; and cases with missing outcome data were excluded outright. Continuous variables were retained in their original form—without truncation or categorization—and not log-transformed (to preserve clinical interpretability in the nomogram). Categorical variables were converted to dummy variables before modeling.

Univariate Cox regression screened variables associated with cardiovascular events (P < 0.20 retained). LASSO-penalized Cox regression with 10-fold cross-validation then performed dimensionality reduction, retaining only variables with non-zero coefficients. These LASSO-selected variables entered multivariable Cox regression to identify independent predictors; forest plots displayed univariate and multivariate results. The proportional hazards assumption for all final-model variables was verified using the Schoenfeld residual test. Discriminative ability of individual univariate predictors was preliminarily evaluated using time-dependent receiver operating characteristic (ROC) analysis, while Kaplan–Meier (KM) curves with log-rank tests assessed their prognostic separation capacity.

The final prediction model was developed and internally validated via bootstrap resampling (500 iterations), using a prespecified 7:3 training–validation split. Model outputs were translated into an intuitive, clinically interpretable nomogram. The optimism-corrected C-index was calculated across all 500 repeated split rounds to eliminate overfitting-derived optimistic bias, which served as the primary metric for evaluating the discriminative performance of the nomogram. To evaluate predictive accuracy, time-dependent ROC analysis was performed at prespecified horizons (3-, 5-, and 8-year CVD), and the AUC was calculated with 95% CIs. Optimal risk stratification thresholds were determined using Youden’s index on the 3-year ROC curve; patients were then dichotomized into low- and high-risk groups, and KM curves with log-rank testing were used to compare cumulative MACE incidence. Calibration was assessed graphically (calibration plots) and quantitatively (Harrell’s C-index and calibration slope), evaluating agreement between predicted probabilities and observed event rates. DCA was used to evaluate the clinical benefits and utility of treating patients at risk of MACE predicted by the model. R software version 4.3.1 was used for the entire process of nomogram establishment and validation. A two-tailed test was used in statistical analysis, with a significance level of α = 0.05; P < 0.05 was considered statistically significant.

## Results

A total of 559 patients who underwent new catheter insertion and initiated regular PD were enrolled. Of these, 123 patients were excluded: 38 had a dialysis duration of less than three months, 26 had pre-dialysis CVD, 42 were lost to follow-up, 9 had a history of kidney transplantation, and 8 were under 18 years of age. Ultimately, 436 PD patients meeting the inclusion criteria were included in the analysis. Among them, 132 deaths occurred during follow-up; of these, 70 (53.0%) were attributable to MACE. The mean follow-up duration was 48.25 ± 24.05 months. The cohort comprised 182 male patients (41.7%) and 254 female patients (58.3%), with a mean age of 48.12 ± 13.76 years. Glomerulonephritis was the most common primary kidney disease (298 cases, 68.5% of the total PD cohort), followed by diabetic nephropathy and hypertensive renal damage.

Univariate analyses were performed with MACE as the primary outcome and relevant clinical, biochemical, and echocardiographic variables as covariates. Significant between-group differences were observed in age, presence of edema, underlying primary renal disease, albumin, prealbumin, albumin-to-globulin ratio, fasting blood glucose(FBG), sodium, chloride, parathyroid hormone, absolute and relative handgrip strength, left ventricular end-diastolic diameter(LVEDD), left ventricular fractional shortening(LVFS) and left ventricular ejection fraction(LVEF) (P < 0.05). No statistically significant differences were detected in other baseline characteristics, laboratory parameters, or echocardiographic indices (P > 0.05). Although median N-terminal pro-B-type natriuretic peptide(NT-proBNP) levels were numerically higher in the MACE group compared with the non-event group, this difference did not reach statistical significance (P = 0.130) (**Table 1**).

**Table 1:** Comparison of baseline data between the MACE group and the non- MACE group.

Item	Total(n=436)	Without MACE (n=366)	With MACE (n=70)	t/z/χ^2^ Value	P Value
Sex (male, %)	182(41.7%)	152(41.5%)	30(42.9%)	0.043	0.837
Age (years)	48.12 ± 13.76	46.45 ± 13.49	56.84 ± 11.80	-6.017	<0.001
Smoking (%)	149(34.2%)	123(33.6%)	26(37.1%)	0.936	0.587
Height (cm)	167(160,172)	165.95 ± 7.90	165.03 ± 8.35	0.890	0.374
Weight (kg)	62.01 ± 11.73	62.05 ± 11.62	61.82 ± 12.40	0.148	0.882
BMI (kg/m^2^)	22.47 ± 3.48	22.45 ± 3.42	22.61 ± 3.82	-0.343	0.732
SBP (mmHg)	130(130,150)	130(120,150)	115(110,130)	-0.185	0.854
DBP (mmHg)	80(75,100)	80(75,90)	80(72.25,90)	-0.997	0.319
Edema (Present, %)	99(22.7%)	76(17.4%)	23(5.3%)	8.091	0.044
Primary renal disease				8.939	0.030
Chronic glomerulonephritis	298(68.5%)	260(71.0%)	38(54.3%)		
Diabetic nephropathy	79(18.1%)	63(17.2%)	16(22.9%)		
Hypertensive renal damage	35(8%)	25(6.8%)	10(14.3%)		
Others	28(6.4%)	18(5.0%)	6(8.5%)		
Peritoneal transport function (%)				0.794	0.851
Low transport	69(15.8%)	60(16.4%)	9(12.9%)		
Low average transport	192(44.0%)	160(43.7%)	32(45.7%)		
High average transport	139(31.9%)	115(31.4%)	24(34.3%)		
High transport	36(8.3%)	31(8.5%)	5(7.1%)		
Hb (g/L)	105.28 ± 17.95	105.89 ± 17.77	102.11 ± 18.69	1.614	0.107
PLT (10^9^/L)	174(130.75,217)	173.5(133.75,218)	174.5(125,204.5)	-0.632	0.527
WBC (10^9^/L)	6.29(4.9,8.01)	6.25(4.87,8.01)	6.5(5.01,8.04)	-0.589	0.556
CRP (mg/L)	1.45(0.52,4.28)	1.38(0.5, 3.75)	2.13(0.61,5.51)	-1.650	0.099
NT-proBNP (pg/mL)	782.5(251,3576.5)	784.5(247.5,2665.75)	745(256,4641)	-1.581	0.130
Albumin (g/L)	37.39 ± 5.04	37.69 ± 4.93	35.85 ± 5.36	2.816	0.005
Prealbumin (mg/L)	376.78 ± 89.084.14	380.75(326.63,437.98)	365.95(293.25,404.05)	-2.082	0.037
A/G ratio	1.3(1.1,1.5)	1.30(1.15,1.55)	1.21(1.00,1.43)	-2.775	0.006
TG (mmol/L)	1.74(1.23,2.48)	1.74(1.24,2.52)	1.73(1.09,2.36)	-0.531	0.596
TyG index	1.62 ± 0.68	1.60 ± 0.69	1.72 ± 0.63	-1.415	0.158
TC (mmol/L)	4.47(3.86,5.79)	4.44(3.86,5.11)	4.68(3.90,512)	-0.935	0.350
HDL-C (mmol/L)	1.03(0.84,1.28)	1.09 ± 0.37	1.13 ± 0.34	-0.870	0.385
LDL-C (mmol/L)	2.70(2.09,3.20)	2.67(2.09,3.21)	2.76(2.14,3.19)	-0.374	0.708
Phosphorus (mmol/L)	1.37(1.15,1.62)	1.41 ± 0.38	1.45 ± 0.36	-0.813	0.417
Chloride (mmol/L)	99.6(96.6,102.7)	99.70(97.10,102.73)	98.05(94.88,101.25)	-2.362	0.018
Magnesium (mmol/L)	0.96(0.86,1.06)	0.96(0.87,1.06)	0.92(0.84,1.05)	-1.524	0.127
Sodium (mmol/L)	142(140,144)	142.07 ± 3.21	141.22 ± 3.63	1.989	0.047
Potassium (mmol/L)	4.14 ± 0.73	4.16 ± 0.69	4.07 ± 0.90	0.907	0.365
Calcium (mmol/L)	2.12 ± 0.21	2.13 ± 0.20	2.11 ± 0.24	0.683	0.495
FBG (mmol/L)	5.11(4.63,6.23)	4.98(4.56,5.91)	5.97(5.41,7.45)	-6.303	<0.001
Fibrinogen (g/L)	4.55(3.72,5.45)	4.78 ± 1.62	496 ± 1.40	-0.910	0.364
BUN (mmol/L)	18.21 ± 5.19	18.17 ± 5.20	18.39 ± 5.15	-0.325	0.745
Scr (umol/L)	790.17 ± 267.84	787.95 ± 260.44	801.78 ± 305.45	-0.395	0.693
SUA (umol/L)	367.75 ± 71.31	367.86 ± 72.06	367.20 ± 67.80	0.070	0.944
PTH (pg/mL)	334.30(215.25,473.33)	344.85(228.43,483.38)	272.05(185.95,393.63)	-3.135	0.002
Absolute handgrip strength (kg)	53.75 ± 16.60	56.05 ± 16.28	41.35 ± 12.43	8.588	<0.001
Relative handgrip strength (kg)	2.43 ± 0.79	2.54 ± 0.77	1.87 ± 0.63	7.713	<0.001
Ultrafiltration volume (ml)	600(300,950)	600(300,950)	600(292.5, 1000)	-0.089	0.929
Net fluid balance (ml)	1335(1000,1615)	1350(1050,1600)	1275(887.5,1642.5)	-1.306	0.192
Urine volume (ml)	723.77 ± 501.06	733.78 ± 490.16	624.40 ± 494.10	1.755	0.080
Urinary urea Kt/V	0.46(0.24,0.74)	0.48(0.25,0.75)	0.37(0.17,0.69)	-1.461	0.144
Dialysate urea Kt/V	1.48 ± 0.42	1.48 ± 0.42	1.46 ± 0.44	0.465	0.642
Total urea Kt/V	2.06 ± 0.54	2.07 ± 0.53	2.03 ± 0.58	0.522	0.602
Urinary creatinine clearance	31.09(14.88,52.57)	32.20(15.94,53.46)	20.37(8.31,50.63)	-1.863	0.062
Dialysate creatinine clearance	41.21 ± 10.82	41.15 ± 10.76	41.54 ± 11.23	-0.280	0.780
Total creatinine clearance	73.70(59.61,96.09)	74.77(59.94,97.28)	69.61(58.22,91.11)	-1.356	0.175
nPCR	0.91(0.80,1.02)	0.91(0.80,1.02)	0.91(0.77,1.01)	-0.017	0.987
LAD (mm)	34.19 ± 5.58	34.02 ± 5.78	35.13 ± 4.29	-1.531	0.127
LVEDD (mm)	52.06 ± 6.81	51.76 ± 6.58	53.73 ± 7.75	-1.990	0.050
LVESD (mm)	33.75 ± 6.73	33.52 ± 6.55	34.92 ± 7.58	-1.605	0.109
IVST (mm)	9.81 ± 2.45	9.90 ± 2.54	9.40 ± 1.84	1.571	0.117
LVPWT (mm)	9(8,11)	9(8,11)	8(8,11)	-1.541	0.123
RVAD (mm)	16(15,17)	16(15,17)	17(15.75,18)	-1.873	0.061
LVEF (%)	64(59,69)	63.77 ± 8.30	58.95 ± 12.00	3.213	0.002
LVFS (%)	34.70 ± 6.48	35.22 ± 5.89	31.97 ± 8.48	3.069	0.003

MACE, major adverse cardiovascular events; Hb, hemoglobin; SBP, Systolic blood pressure; DBP, Diastolic blood pressure; CRP, C-reactive protein; A/G rati, Albumin/Globulin ratio; TG, Triglyceride; TyG index, Triglyceride-glucose index; TC, Total cholesterol; HDL-C, How-density lipoprotein cholesterol; LDL-C, Low-density lipoprotein cholesterol; BUN, Blood urea nitrogen; Scr, Serum creatinine (μmol/L); SUA, Serum uric aci; PTH, Parathyroid hormone; nPCR, Normalized protein catabolic rate; LAD, Left atrial anteroposterior diameter; LVESD, Left ventricular end-systolic diameter; IVST, Interventricular septal thickness; LVPWT, Left ventricular posterior wall thickness; RVAD, Right ventricular anteroposterior diameter.

**Figure 1:**
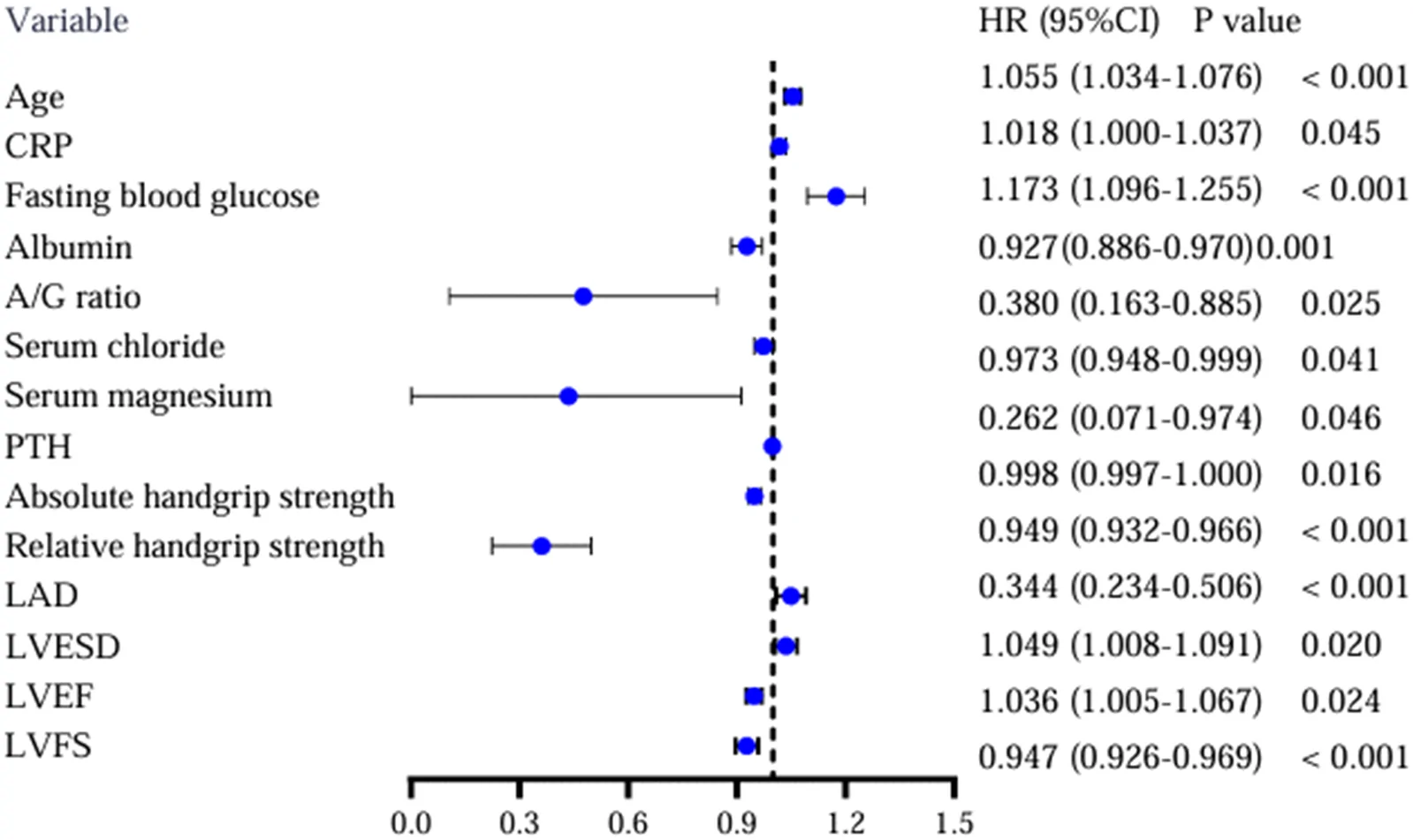
Forest plot of univariate COX regression for predicting MACE in peritoneal dialysis patients.

Univariate Cox regression analysis of all variables revealed that age (HR = 1.054, 95% CI: 1.03–1.076; P < 0.001), CRP (HR = 1.018, 95% CI: 1.000–1.037; P = 0.045), FBG (HR = 1.173, 95% CI: 1.096–1.255; P < 0.001), ALB(HR = 0.927, 95% CI: 0.886–0.970; P = 0.001), A/G ratio (HR = 0.380, 95% CI: 0.163–0.885; P = 0.025), chloride (HR = 0.973, 95% CI: 0.948–0.999; P = 0.041), magnesium (HR = 0.262, 95% CI: 0.071–0.974; P = 0.046), PTH (HR = 0.998, 95% CI: 0.997–1.000; P = 0.016), absolute handgrip strength (HR = 0.949, 95% CI: 0.932–0.966; P < 0.001), relative handgrip strength (HR = 0.344, 95% CI: 0.234–0.506; P < 0.001), LAD (HR = 1.049, 95% CI: 1.008–1.091; P = 0.020), LVESD (HR = 1.036, 95% CI: 1.005–1.067; P = 0.024), LVEF (HR = 0.947, 95% CI: 0.926–0.969; P < 0.001), and LVFS (HR = 0.929, 95% CI: 0.898–0.961; P < 0.001) were all statistically significant predictors (P < 0.05) (**Figure 1**).

**Figure 2:**
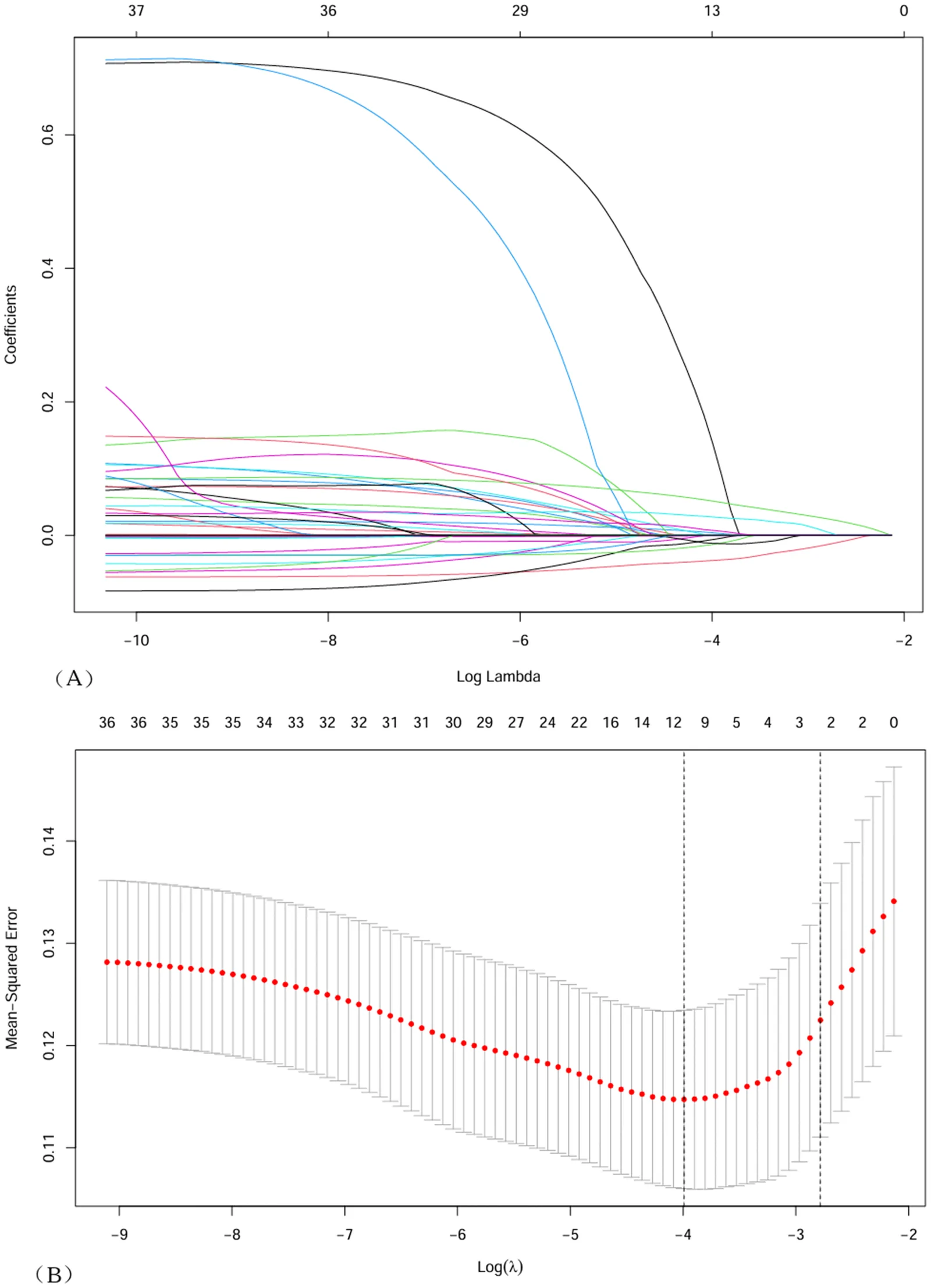
LASSO coefficients of MACE in peritoneal dialysis patients. **(a)** Each curve in the figure represents the change of each variable in the coefficients. **(b)** The figure shows the 10-fold cross-validation fit.

Lasso regression analysis incorporated 37 MACE prognosis–related factors reported in the literature. Using λ = 0.0185—the value corresponding to the minimum cross-validation deviance—12 predictors were selected: sex, age, hemoglobin, FBG, lymphocyte count, triglyceride, serum creatinine concentration, PTH, absolute handgrip strength, LVEDD, LVESD, LVEF, LVFS. Multicollinearity diagnostics confirmed the absence of substantial collinearity among these 12 variables. Further applying λ = 0.0619—the value corresponding to one standard deviation above the minimum deviance—yielded a more parsimonious model comprising three key predictors: age, absolute handgrip strength, and LVEF (**Figure 2**).

**Figure 3:**
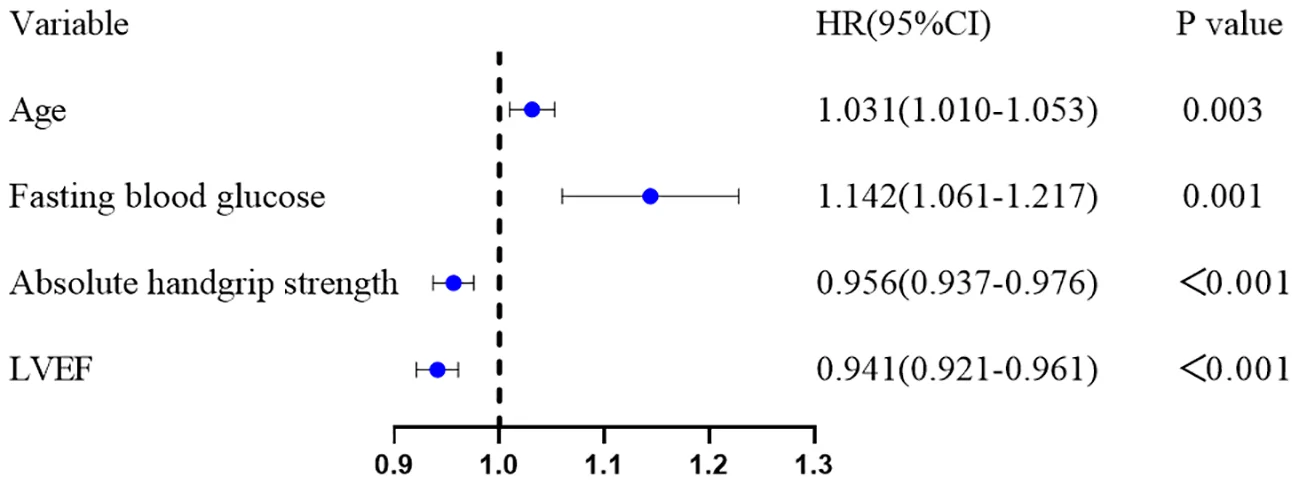
Forest plot of multivariate COX regression for predicting MACE in peritoneal dialysis patients.

Variables exhibiting statistically significant differences in univariate analysis were subsequently entered into multivariate Cox proportional hazards regression. The multivariate analysis identified age (HR = 1.031; 95% CI: 1.010–1.053; P = 0.003), FBG (HR = 1.142; 95% CI: 1.061–1.217; P < 0.001), absolute handgrip strength (HR = 0.956; 95% CI: 0.937–0.976; P < 0.001), and LVEF (HR = 0.941; 95% CI: 0.921–0.961; P < 0.001) as independent prognostic factors for MACE among patients undergoing PD (**Figure 3**).

**Figure 4:**
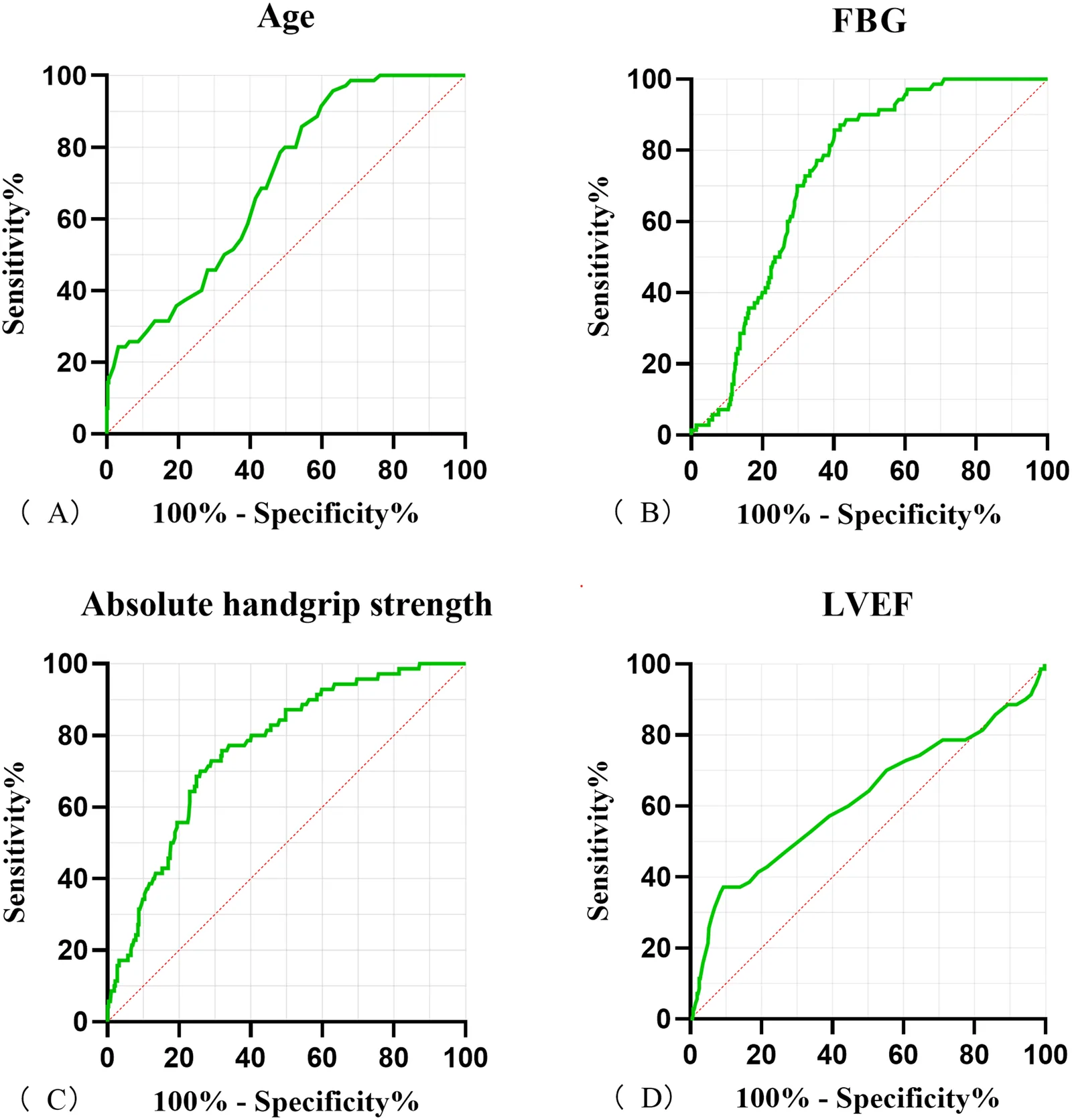
Receiver operating characteristic curves of age **(a)**, fasting blood glucose **(b)**, absolute grip strength **(c)**, and left ventricular ejection fraction **(d)**.

ROC curves were constructed using four variables: age, FBG, absolute handgrip strength, and LVEF. The corresponding AUC values were 0.698 (95% CI: 0.639–0.758), 0.738 (95% CI: 0.688–0.788), 0.763 (95% CI: 0.706–0.819), and 0.619 (95% CI: 0.537–0.701), respectively. FBG and absolute handgrip strength demonstrated good predictive discrimination (AUC > 0.7), whereas age and LVEF exhibited moderate predictive discrimination (AUC > 0.6) (**Figure 4**).

**Figure 5:**
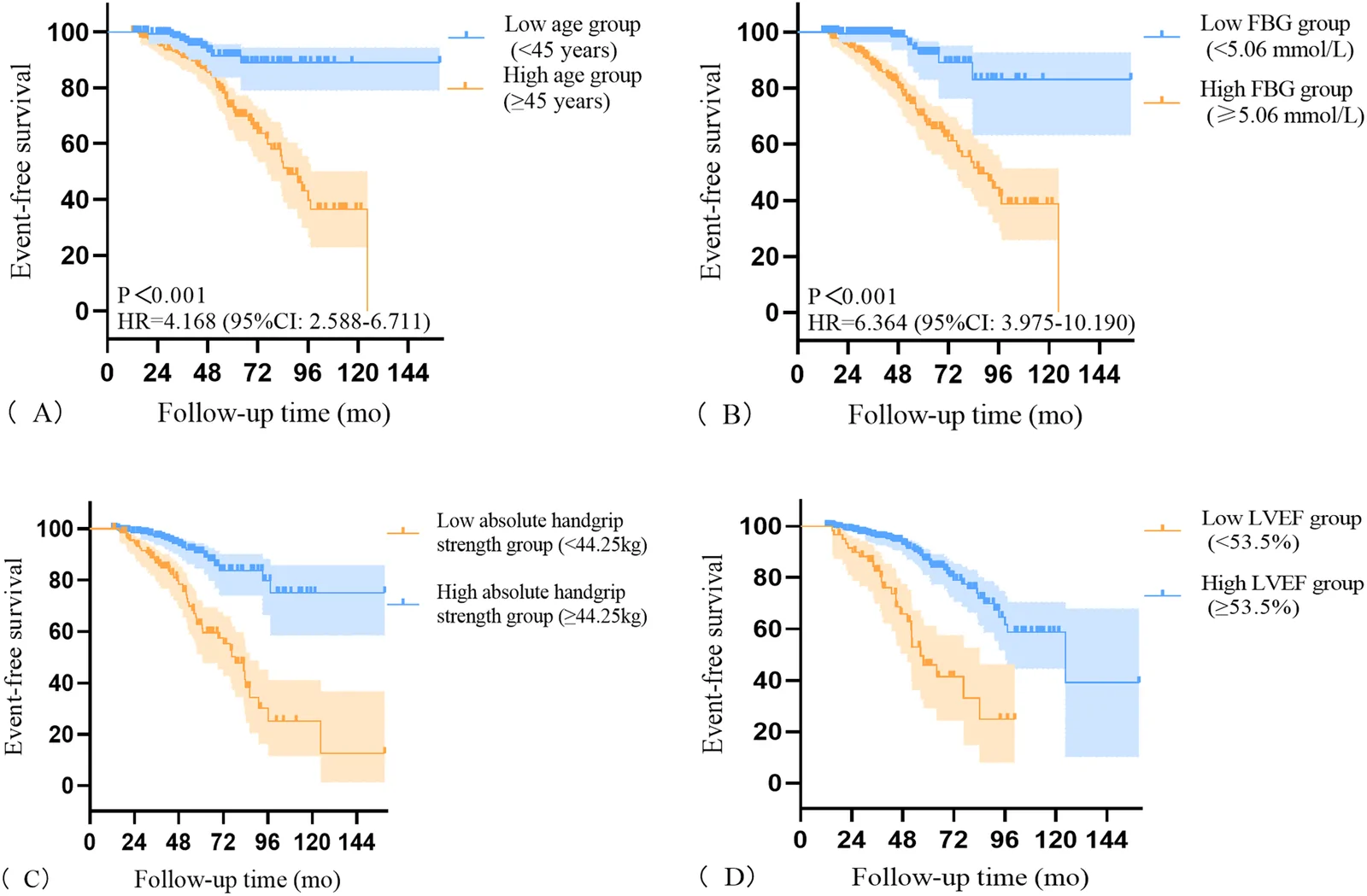
Comparison of survival rates for MACE (Kaplan-Meier curves).

The optimal cutoff values were derived from the ROC presented in **Figure 4**, and patients were subsequently stratified into high-risk and low-risk groups based on these thresholds. Survival outcomes across risk groups were compared using KM curves. As shown in **Figure 5**, multivariable Cox regression analysis identified age, FBG, absolute handgrip strength, and LVEF as independent predictors of MACE among patients undergoing PD (P < 0.05).

**Figure 6:**
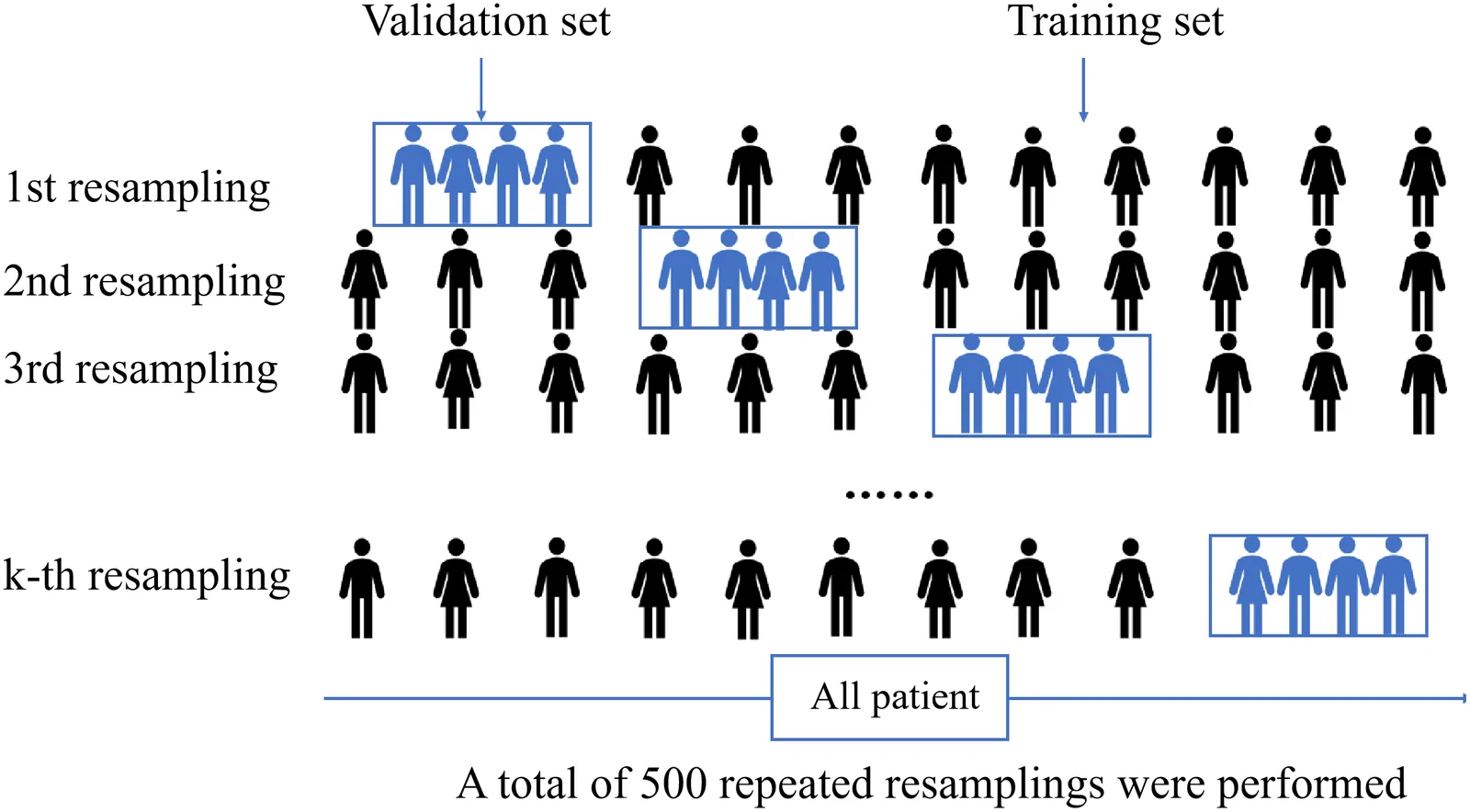
Bootstrap resampling.

**Figure 7:**
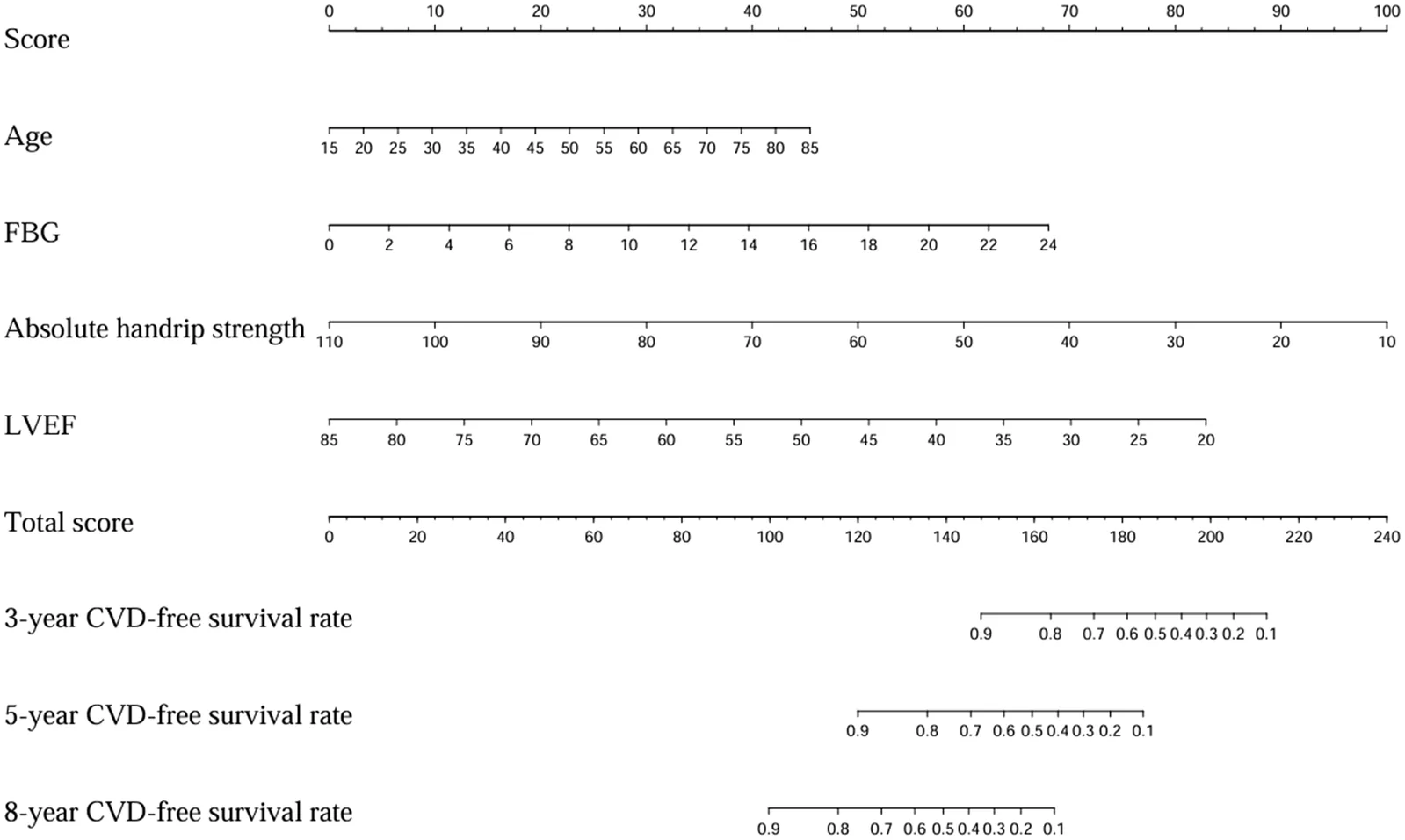
Nomogram for predicting the survival rate without MACE in peritoneal dialysis patients. All raw glycemic data were retained for model fitting without exclusion; extreme high FBG values were exclusively from participants with long-standing poorly controlled diabetes mellitus.

By integrating variables identified through both LASSO regression and multivariate Cox regression, the final prediction model incorporated four key covariates: age, FBG, absolute handgrip strength, and LVEF. The model was developed using the bootstrap resampling method with 500 iterations (**Figure 6**). Given the relatively small regularization coefficient (i.e., the tuning hyperparameter) employed in this study, a 7:3 training–validation split was applied in each bootstrap resample. After 500 rounds of repeated hold-out validation, three model discrimination metrics were derived: the C-index (equivalent to AUC) computed on the full dataset was 0.8277; the mean C-index (AUC) across all 70% training subsets was 0.8717; and the mean C-index (AUC) on the corresponding 30% held-out validation subsets was 0.7290. A nomogram was constructed to predict MACE–free survival among PD patients, based on the selected variables. Specifically, this nomogram enables estimation of 3-, 5-, and 8-year MACE–free survival probabilities as functions of age, FPG, absolute handgrip strength, and LVEF (**Figure 7**). Calibration and contribution analyses revealed that absolute handgrip strength exerted the strongest prognostic influence, followed by LVEF, FPG, and age.

**Figure 8:**
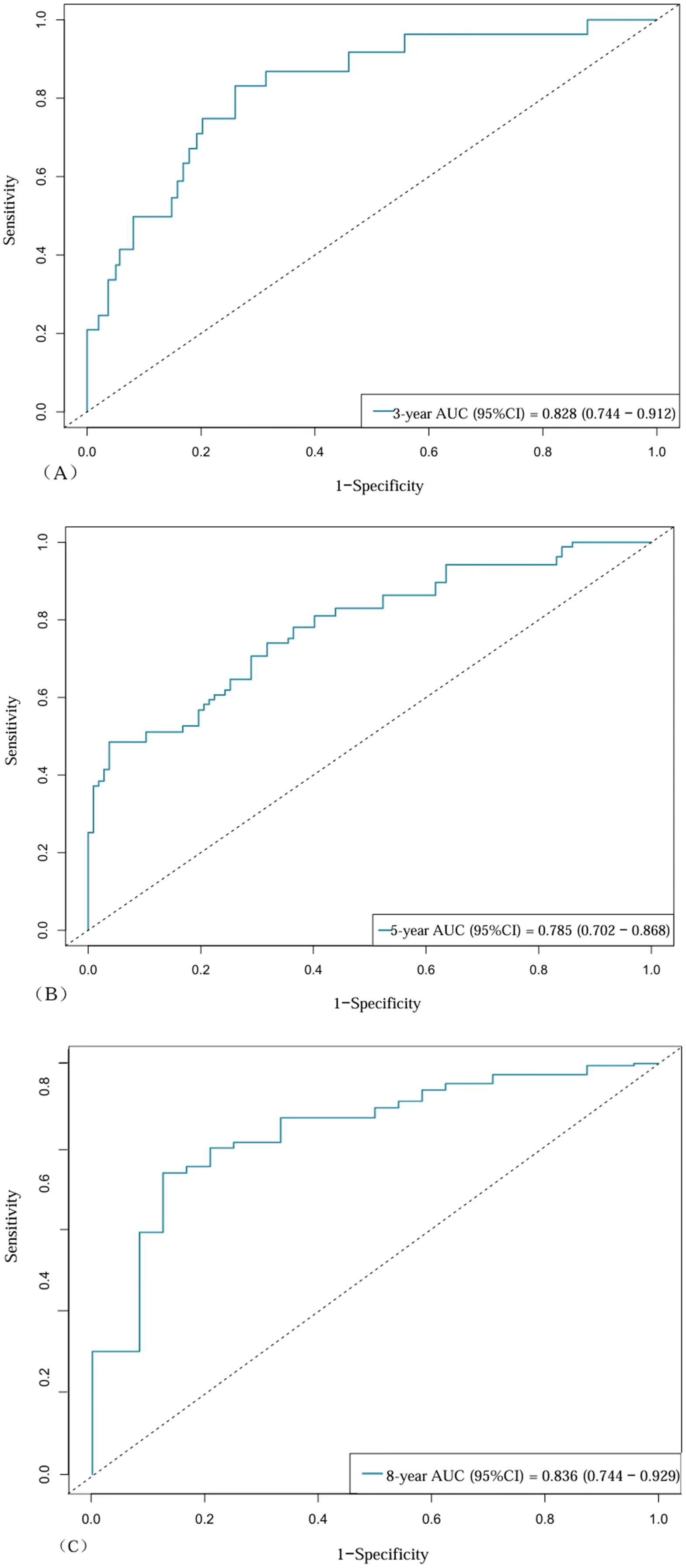
The ROC and AUC of the predictive model.

The nomogram demonstrated AUC values of 0.828 (95% CI: 0.744–0.912), 0.785 (95% CI: 0.702–0.868), and 0.836 (95% CI: 0.744–0.929) for predicting 3-, 5-, and 8-year MACE–free survival in PD patients, respectively (**Figure 8**). These findings indicate that the prediction model exhibits robust discriminative performance.

**Figure 9:**
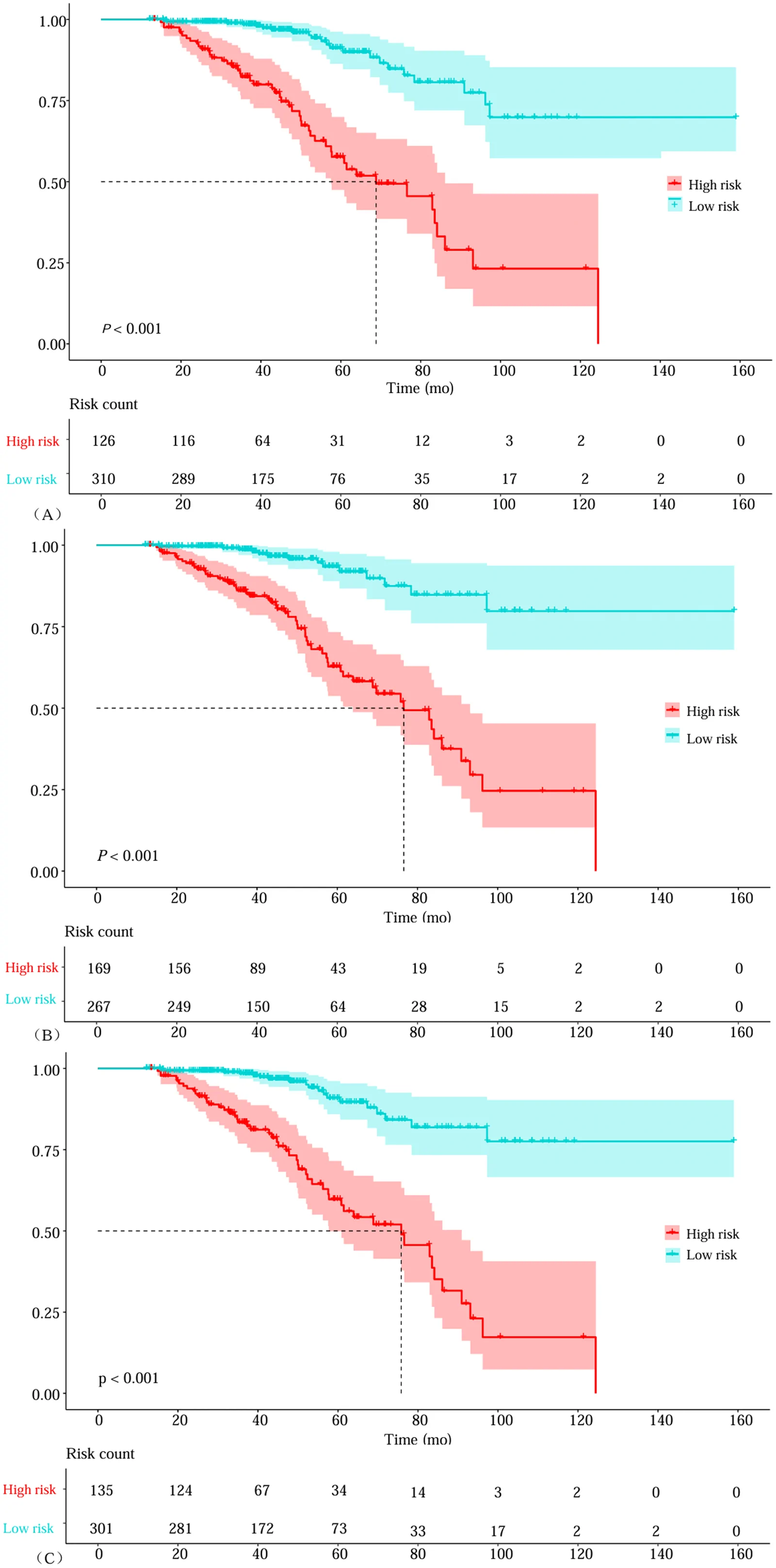
After the nomogram scoring generated the risk stratification model, Kaplan-Meier curves were plotted to compare the cumulative incidence of MACE at 3 years **(a)**, 5 years **(b)**, and 8 years **(c)**.

A risk stratification model was developed based on each patient’s total score derived from the nomogram and the optimal cutoff value determined by ROC analysis. Patients were subsequently categorized into low-risk and high-risk groups. KM curves for 3-, 5-, and 8-year overall survival were generated separately for each group. The results demonstrated statistically significant differences in the cumulative incidence of MACE at all three time points (P < 0.001; **Figure 9**), indicating that the prediction model exhibits excellent discriminative performance.

**Figure 10:**
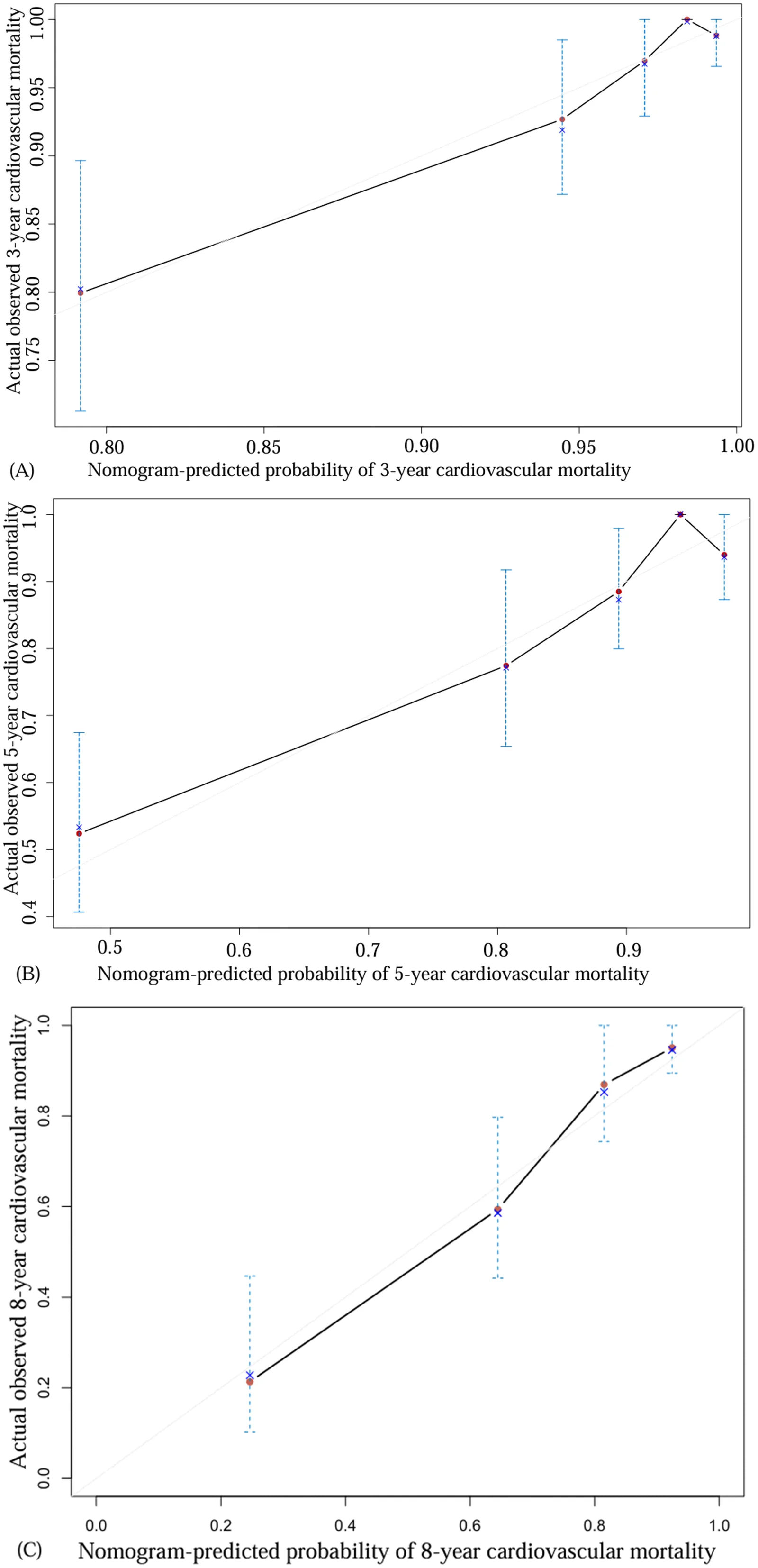
Calibration plot of the actual probability of MACE in peritoneal dialysis patients versus the predicted probability by the nomogram.

In the calibration curves, the horizontal axis denotes the predicted probability of MACE among PD patients, while the vertical axis represents the corresponding observed (empirical) probability of such events in this population. The diagonal reference line (i.e., the 45°line) signifies perfect calibration—where predicted probabilities align exactly with observed event frequencies. As illustrated in **Figure 10**, the calibration curves across all evaluated time points closely approximate this ideal diagonal line, indicating good overall agreement between predicted and observed MACE probabilities.

**Figure 11:**
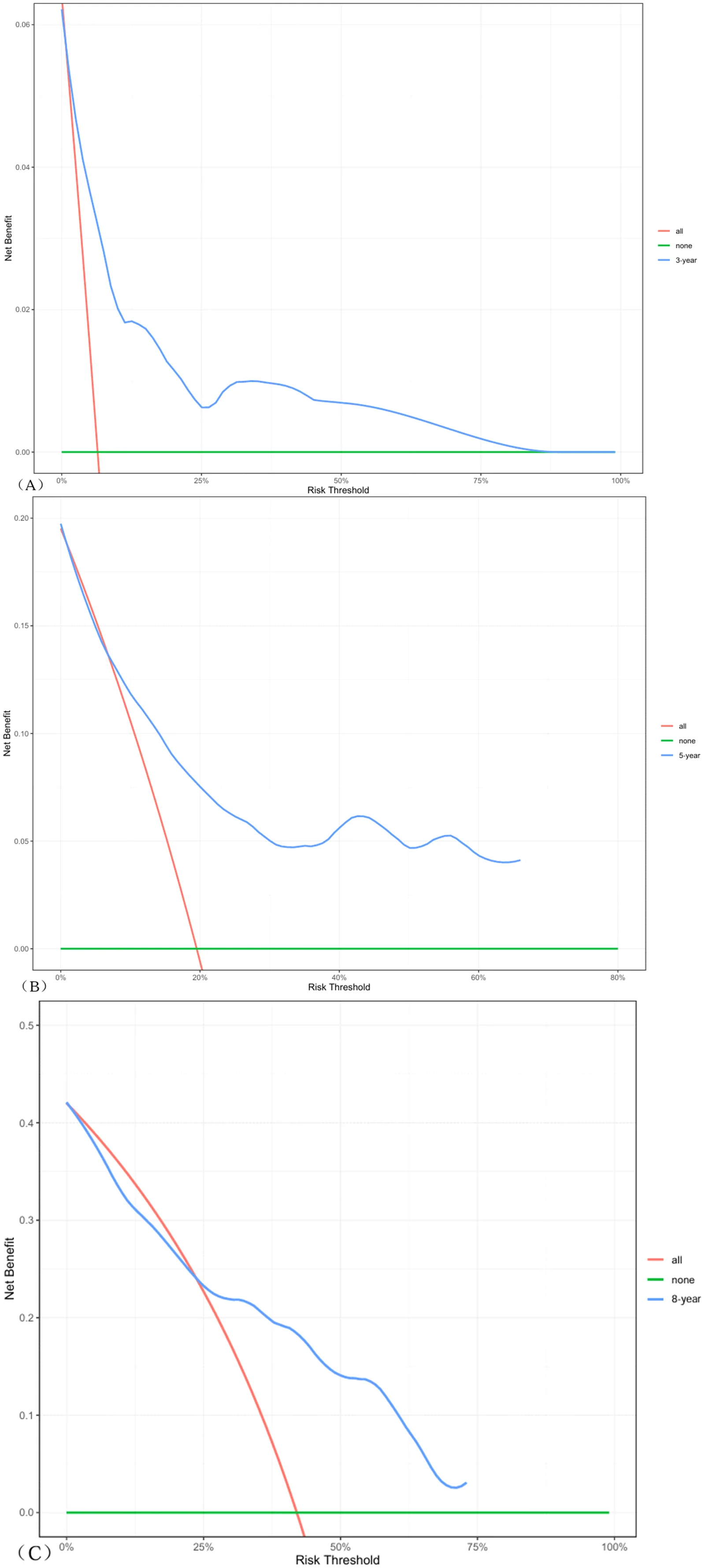
Decision curve analysis of the occurrence of MACE in peritoneal dialysis patients at 3 years **(a)**, 5 years **(b)**, and 8 years **(c)** in the predictive model.

DCA was conducted to assess the clinical utility of the nomogram for predicting MACE. For 3-year MACE, the model-derived risk probabilities demonstrated favorable net benefit across a broad range of threshold probabilities (**Figure 11a**). For 5-year events, no significant net benefit advantage was observed for selectively intervening in high-risk patients—compared with managing the entire population—when the threshold probability ranged from 0% to 8%; however, selective intervention in high-risk patients yielded greater net benefit than either no intervention or universal intervention when the threshold probability exceeded 8% (**Figure 11b**). For 8-year events, the net benefit of selectively intervening in high-risk patients was marginally lower than that of universal intervention at threshold probabilities below 22%, but became superior when the threshold probability exceeded 22% (**Figure 11c**). Collectively, DCA indicated robust overall clinical utility of the prediction model.

## Discussion

CVD is the leading cause of elevated morbidity and mortality among patients undergoing long-term dialysis. In our study, 70 cardiovascular deaths occurred—representing 16% of the total cohort and 53% of all-cause mortality—consistent with prior reports indicating a MACE incidence of approximately 15% (5) and cardiovascular deaths accounting for roughly 50% of total mortality in PD patients (6). Beyond conventional cardiovascular risk factors—including advanced age, smoking, hypertension, diabetes mellitus, and hypercholesterolemia—chronic glucose absorption during long-term PD and progressive accumulation of uremic toxins secondary to declining renal function further accelerate MACE progression in this population. To improve survival outcomes, we developed a prognostic model specifically designed to identify PD patients at high risk for MACE. Univariate and multivariate Cox proportional hazards regression analyses identified age, FBG, absolute handgrip strength, and LVEF as independent predictors of cardiovascular death. Based on these four baseline variables, we constructed a novel nomogram that demonstrated favorable predictive accuracy and discrimination, along with strong clinical utility and generalizability.

Physiological aging leads to concentric myocardial hypertrophy, progressive impairment of diastolic function, diminished systolic reserve, increased stiffness of the aorta and large arteries, and impaired endothelium-dependent vasodilation—collectively forming the pathophysiological foundation of CVD in older adults (7). These age-associated structural and functional changes are closely linked to metabolic alterations, endothelial cell senescence, immune dysregulation, and telomere attrition (8). In this study, PD patients who experienced MACE had a significantly higher mean age than those who remained event-free. Multivariate Cox proportional hazards regression analysis identified age as an independent predictor of MACE risk among PD patients. Consistent with our findings, prior studies have demonstrated that elderly PD patients face a substantially elevated risk of MACE compared with their younger counterparts (9). This heightened susceptibility is partly attributable to age-related increases in oxidative stress, which drive chronic inflammation and promote myocardial fibrosis (10).

Poor nutritional status constitutes a significant risk factor for CVD among patients with CKD (11). The prevalence of malnutrition reaches 59% in PD patients with a history of heart failure—substantially higher than in those without underlying congestive heart failure (12). Although malnutrition is rarely a direct cause of cardiovascular mortality, it may impair cardiac contractility, promote the development and progression of atherosclerotic vascular disease, and exacerbate pre-existing CVD, thereby contributing to fatal outcomes (13). These adverse effects may be mediated, at least in part, by sarcopenia—a condition characterized by loss of skeletal muscle mass and function—which facilitates intramuscular and visceral fat accumulation, both of which are independently associated with increased CVD risk (14). Furthermore, pro-inflammatory cytokines—including TNF-α, IL-1, and IL-6 (15)—drive muscle protein catabolism in CKD patients and exert detrimental effects on myocardial structure and function. Patients with heart failure or other catabolic conditions frequently experience reduced exercise capacity secondary to mobility-limiting skeletal muscle atrophy (16), thereby accelerating the decline in muscle strength. Notably, prior evidence indicates that muscle strength—not muscle mass—is a more robust predictor of nutritional status in this population (17); thus, identifying modifiable factors influencing muscular strength is critical for effective malnutrition management. Handgrip strength, a simple, safe, non-invasive, radiation-free, and cost-effective tool for nutritional assessment, serves as a reliable surrogate marker for lean body mass(LBM) and has been shown to predict survival and mortality in patients with ESRD (18). In the present study, PD patients who experienced MACE exhibited significantly higher absolute and relative handgrip strength values compared with those who remained event-free. Univariate Cox proportional hazards regression analyses revealed strong associations between both handgrip strength indices and incident MACE. These findings align with previous reports demonstrating robust correlations between handgrip strength and both all-cause and cardiovascular mortality (19). Collectively, these results underscore the potential clinical utility of handgrip strength as a biomarker for cardiovascular risk stratification in PD patients and warrant further investigation into its role in CVD assessment and management.

Some studies suggest that relative handgrip strength may be superior to absolute handgrip strength in predicting MACE and other cardiometabolic disorders (20). However, in our study, absolute handgrip strength remained a significant independent predictor of MACE among PD patients—even after adjustment for other relevant confounders—whereas the predictive value of relative handgrip strength diminished substantially. This discrepancy may stem from inconsistent associations between BMI and handgrip strength across populations. For instance, a cross-sectional analysis from the Health, Aging and Body Composition Study reported an inverse relationship between BMI and both handgrip strength and LBM (21). In contrast, a meta-analysis of UK cohort studies found that handgrip strength increased with higher BMI in men but showed no statistically significant differences across BMI categories in women (22). Such inconsistency likely reflects fundamental limitations of BMI as a proxy for adiposity: BMI incorporates LBM—a key determinant of muscular strength—alongside fat mass, thereby conflating components with opposing physiological influences. Moreover, BMI fails to capture regional fat distribution, which differentially impacts health outcomes; for example, peripheral (e.g., limb) adiposity may support or preserve muscle function, whereas central obesity is associated with systemic inflammation, insulin resistance, and adverse cardiovascular effects. Supporting this notion, Keevil et al.’s UK-based study in middle-aged and older men demonstrated that each 10-cm increase in waist circumference was associated with a 3.56-kg reduction in handgrip strength (23). Collectively, these findings suggest that the heterogeneous and context-dependent relationship between BMI and handgrip strength may underlie the superior prognostic performance of absolute—rather than BMI-adjusted (i.e., relative)—handgrip strength in our cohort.

In non-diabetic patients, continuous absorption of peritoneal dialysate glucose causes glucose metabolism disorders, insulin resistance, and increased lipogenesis, leading to higher all-cause and cardiovascular mortality in PD patients than in HD patients (24). Insulin resistance is a key factor increasing MACE risk in these patients (25). A large prospective cohort study found insulin resistance surrogate markers (e.g., plasma triglycerides, glucose) were associated with cardiovascular mortality (26). FBG, a serological indicator of diabetes or dialysate glucose exposure, is an independent predictor of MACE (27). In our cohort, f FBG correlated with MACE in both univariate and multivariate analyses. Elevated FBG vascular endothelial cells, promotes smooth muscle cell migration/proliferation, activates plasminogen activator inhibitor-1, and enhances procoagulant/antithrombotic factor activity, accelerating thrombosis and MACE (28). Additionally, inflammatory cytokines in PD patients induce cytotoxicity, pancreatic β-cell apoptosis, and increased insulin resistance (29). The FBG threshold for adverse cardiovascular prognosis is 5.6 mmol/L (30), consistent with our finding that glucose >5.06 mmol/L increases cardiovascular mortality. In summary, FBG, as a simple insulin resistance surrogate, independently predicts MACE in PD patients.

In patients with CKD, hypoalbuminemia may arise from multiple interrelated factors, including inadequate nutritional intake, altered protein catabolism, diminished protein anabolism, chronic systemic inflammation, and losses associated with dialysis therapy. Low serum albumin concentration is a hallmark of protein-energy wasting (PEW) and serves as an established risk factor for MACE among individuals undergoing PD. Notably, hypoalbuminemia at PD initiation constitutes an independent predictor of subsequent MACE and reduced all-cause mortality (31). Additionally, urinary and peritoneal dialysate protein losses are independently associated with elevated risks of MACE and acute cerebrovascular disease (32). ALB also functions as a sensitive biomarker of systemic inflammation, given its well-documented downregulation during inflammatory states. This biological link may partly explain why serum albumin did not emerge as an independent predictor of MACEs in our analysis—its apparent association with MACE may be mediated, at least in part, by underlying inflammatory activity. Moreover, ALB exhibit substantial interindividual and intraindividual variability, influenced by fluctuations in nutritional status, inflammatory burden, and dialysate protein losses; consequently, baseline albumin measurements may hold less prognostic value than longitudinal trends or dynamic changes over time.

Over the past several years, brain natriuretic peptide (BNP) and NT-proBNP have exerted a substantial influence on cardiovascular research and the clinical management of heart disease (33); however, their routine clinical application remains limited. Both BNP and NT-proBNP exhibit age-dependent variations, with aging potentially altering their synthesis, secretion, or clearance (34). Notably, current clinical reference thresholds for NT-proBNP do not account for age, contributing to frequent misdiagnosis of heart failure in elderly patients—particularly due to age-related elevations in circulating NT-proBNP concentrations. Circulating NT-proBNP levels are inversely associated with BMI (35); although the precise underlying mechanism remains unclear, one proposed explanation is that adipose tissue enhances BNP clearance and attenuates ventricular BNP release (36). Furthermore, hyperglycemia significantly modulates NT-proBNP concentrations in patients with heart failure: prior evidence indicates that diabetes mellitus markedly lowers NT-proBNP, likely through hyperglycemia- and insulin resistance–mediated increases in NT-proBNP glycosylation, thereby impairing its secretion (37). Gender also appears to influence NT-proBNP, possibly attributable to sex hormone–related differences in cardiac neurohormonal regulation (38). Collectively, the confounding effects of age, BMI, sex, and renal function on NT-proBNP obscure clinically meaningful differences—indeed, no statistically significant difference in NT-proBNP levels was observed between PD patients with and without MACE.

Univariate analysis linked lower serum magnesium levels to increased MACE in peritoneal dialysis patients. Prior studies associate hypomagnesemia with higher all-cause and cardiovascular mortality in CKD patients (39). First, magnesium regulates cardiac function by stabilizing myocardial diastolic electrical activity; elevated extracellular magnesium lowers intracellular calcium and suppresses angiotensin-induced aldosterone synthesis, reducing blood pressure (40). Second, magnesium lowers LDL and triglycerides while raising HDL—via enhanced lipoprotein lipase activity and inhibition of HMG-CoA reductase, the rate-limiting enzyme in cholesterol synthesis (41). Third, low serum magnesium in peritoneal dialysis patients may promote vascular calcification, likely through magnesium deficiency–induced disruption of calcium-phosphate metabolism and associated dysregulation of calcium, phosphate, and cytokines (42). Finally, magnesium modulates endothelial function; supplementation improves it in coronary artery disease patients (43). In this study, the association between serum magnesium and MACE weakened after multivariate adjustment. Hypomagnesemia impairs protein synthesis, and malnutrition independently predicts all-cause mortality in dialysis patients (44). Low magnesium may also trigger a pro-inflammatory cascade, suggesting an inverse relationship between serum magnesium and systemic inflammation (45). Magnesium levels interact with most antidiabetic drugs, including SGLT2 inhibitors. In peritoneal dialysis patients, hypomagnesemia arises from multiple factors—malnutrition, inflammation, impaired intestinal absorption, medications, and dialysate loss—all affecting serum magnesium. Consequently, serum magnesium loses predictive value in multivariate analysis when these confounders are accounted for.

This study shows that PTH levels are higher in peritoneal dialysis patients with MACE than in those without. In univariate analysis, PTH predicted MACE, but this association weakened substantially after adjusting for confounders. PTH is essential for calcium and phosphorus homeostasis. Its link with cardiovascular mortality remains inconclusive. Elevated PTH may impair myocardial contractility and promote cardiac hypertrophy, contributing to coronary artery disease and vascular calcification (46). A meta-analysis of 12 cohort studies found that high PTH was associated with a 1.45-fold increased risk of CVD events (95% CI 1.24–1.71) versus low PTH (47). However, a large U.S. prospective study of 79,219 patients across 15 communities found no independent association between elevated PTH and cardiovascular risk (48).

Echocardiography is a widely used, noninvasive imaging modality for evaluating left ventricular (LV) structure and myocardial systolic and diastolic function in patients undergoing PD. It holds established prognostic value for MACE and all-cause mortality in individuals with ESRD. The pathogenesis of LV dysfunction in this population is multifactorial, involving interrelated comorbidities—including volume overload, anemia, uremic toxicity, activation of the renin–angiotensin–aldosterone system, oxidative stress, chronic systemic inflammation, and dysregulated glucose metabolism. LV dysfunction can directly precipitate heart failure and is strongly associated with poor survival, particularly among hypervolemic dialysis patients (49). Moreover, impaired LV systolic function represents a key determinant of cardiovascular symptom burden in this cohort (50). Our study demonstrates that alterations in myocardial contractility—particularly as reflected by LVEF—are clinically critical. LVEF emerged as an independent predictor of MACE in PD patients. A prospective cohort study reported that reduced LVEF was significantly associated with elevated risks of both all-cause and cardiovascular mortality in PD patients (51). Similarly, a Hong Kong–based study found that patients with LVEF < 50% exhibited substantially higher 4-year risks of cardiac death (relative risk [RR] = 2.57) and heart failure hospitalization (RR = 2.25) compared with those with LVEF ≥ 50% (52), corroborating our observation of a potential LVEF threshold—53.5%—beyond which the risk of cardiovascular death increases markedly. Notably, even CKD patients with preserved LVEF may experience adverse outcomes if global LV compliance is compromised (53, 54). Although echocardiography—relying on geometric assumptions to quantify cardiac function—may overestimate LV mass and exhibit measurement variability (55), thereby contributing to inconsistencies across studies, its widespread availability, cost-effectiveness, and intuitive interpretation render it the first-line imaging tool for assessing CKD-related cardiac end-organ damage in routine clinical practice.

Scores for complications, malnutrition, inflammatory status, and vascular calcification have demonstrated utility in predicting cardiovascular risk among dialysis patients. The Malnutrition-Inflammation Score (MIS)—a composite index comprising 10 subjective and objective parameters—is widely debated in clinical practice due to its time-intensive administration and substantial inter-rater subjectivity (56). Similarly, the coronary artery calcification score—assessed via non-contrast cardiac computed tomography—is rarely employed in routine clinical settings owing to concerns regarding ionizing radiation exposure and high associated costs (57). Moreover, PD patients typically exhibit attenuated hemodynamic fluctuations and better-preserved residual renal function compared with HD patients; however, they experience greater albumin loss. Given these distinct pathophysiological profiles, cardiovascular risk factors differ meaningfully between PD and HD populations. Consequently, risk prediction models or scoring systems developed and validated in HD cohorts may lack generalizability to PD patients. The Framingham Risk Score (FRS), the most extensively validated tool for estimating 10-year MACE risk in diverse general populations across multiple ethnic groups, relies exclusively on conventional risk factors. It does not account for dialysis-specific risks—including uremic toxin–mediated myocardial injury, chronic inflammation, or volume overload—rendering it suboptimal for patients with advanced CKD, particularly those undergoing maintenance dialysis. Indeed, a study in dialysis patients reported no statistically significant difference in cardiovascular mortality between FRS-defined high-risk (10-year risk >20%) and low-risk groups (58). In contrast, evidence suggests that augmenting the FRS with PD-relevant biomarkers—specifically serum albumin, hemoglobin, and estimated glomerular filtration rate—significantly improves its predictive performance for MACE in the PD population (59).

Our study developed 3-, 5-, and 8-year MACE risk prediction models specifically for patients undergoing PD. These models incorporated four routinely available, low-cost, time-efficient, and objective clinical variables—age, FBG, absolute handgrip strength, and LVEF-and were visualized as intuitive nomograms, wherein segment length quantitatively reflects each variable’s contribution to the predicted outcome. Absolute handgrip strength emerged as the strongest independent prognostic factor, followed in descending order of importance by LVEF, FBG, and age. Based on model-derived risk scores, patients were stratified into low- and high-risk groups; Kaplan-Meier survival analysis revealed statistically significant differences in MACE-free survival between these groups, demonstrating robust discriminative capacity. Internal validation revealed that the nomogram achieved a higher AUC for predicting cardiovascular event-free survival among peritoneal dialysis patients compared with individual predictors, indicating that the model outperformed single variables in identifying patients at high cardiovascular risk within the present cohort. Decision curve analysis (DCA) preliminarily suggested the potential clinical value of this nomogram. By synthesizing these complementary indicators, the nomogram serves as a practical, evidence-based tool to support clinical decision-making and enable personalized risk management. In summary, we have established a validated, accurate, and clinically translatable nomogram for assessing 3-, 5-, and 8-year risks of MACE in PD patients.

This study has several limitations. First, this is a single-center retrospective cohort study. The inclusion criteria and the single-center sampling source may introduce both recall bias and selection bias. Second, the sample size is insufficient to adequately adjust for all potential confounding variables, thereby limiting the model’s clinical applicability and interpretability. Third, several methodological limitations may affect the validity of the findings: prognostic analyses were based solely on baseline data collected at the initiation of PD; the absence of longitudinal, dynamic follow-up may compromise the generalizability of the predictive model. Furthermore, the lack of detailed medication history, including use of cardioprotective or risk-modifying agents-represents an important gap, as incorporating such information could potentially enhance the accuracy and clinical utility of MACE risk assessment. Fourth, the range of measured biomarkers was limited—key cardiac function indicators such as BNP and cardiac troponins were not included—resulting in a prediction model that omits several biologically plausible predictors and thus cannot achieve absolute predictive precision. Future studies should expand the panel of candidate biomarkers to improve model performance. Finally, although the nomogram’s robustness was rigorously evaluated through extensive internal validation, including repeated Bootstrap resampling-the external generalizability of this tool to PD populations beyond China remains unconfirmed. Accordingly, prospective external validation in diverse, multicenter PD cohorts is warranted.

## Data Availability

The original contributions presented in the study are included in the article/supplementary material. Further inquiries can be directed to the corresponding authors.
