# Supplementary material for: Construction and evaluation of risk prediction model for major adverse cardiovascular events in peritoneal dialysis patients
Source: Front Nephrol. 2026 Sep 3;6:1871318. doi: 10.3389/fneph.2026.1871318 (PMC13581586; doi:10.3389/fneph.2026.1871318)
Supplement: Supplementary file 1 [file Table1.docx]

**Supplementary Table S1**

Baseline characteristics among event-free patients, patients with atherosclerotic thrombotic events, and patients with heart failure/arrhythmia/sudden cardiac death

| **Variable** | **Without MACE (n=366)** | **Atherosclerotic thrombotic events (n=28)** | **HF/Arrhythmia/ SCD (n=42)** | **H/F/χ² Value** | **P Value** |
| --- | --- | --- | --- | --- | --- |
| Sex (male, %) | 152(41.5%) | 13(46.4%) | 17(40.5%) | 0.288 | 0.866 |
| Age (years) | 46.45±13.49 | 58.14±10.82 | 55.98±12.46 | 19.957 | <0.001 |
| BMI (kg/m²) | 22.45±3.42 | 21.90±3.53 | 23.08±3.98 | 1.016 | 0.363 |
| SBP (mmHg) | 130(120,150) | 130.00(122.50,156.25) | 130.00(115.75,140.00) | 1.214 | 0.545 |
| DBP (mmHg) | 80(75,90) | 80.00(74.50,90.00) | 80.00(70.75,90.00) | 1.032 | 0.597 |
| Hb (g/L) | 106.50(97.00,117.00) | 103.50(95.75,112.75) | 103.50(92.50,112.75) | 2.719 | 0.257 |
| CRP (mg/L) | 1.38(0.50,3.75) | 1.54(0.74,4.20) | 2.40(0.58,6.11) | 2.881 | 0.237 |
| Albumin (g/L) | 37.69±4.93 | 35.53±4.78 | 36.07±5.76 | 4.065 | 0.018 |
| Prealbumin (mg/L) | 380.75(326.63,437.98) | 351.69±73.65 | 365.89±91.03 | 1.663 | 0.191 |
| FBG (mmol/L) | 4.98(4.56,5.91) | 5.89(5.42,6.83) | 6.16(5.42,7.40) | 33.706 | <0.001 |
| BUN (mmol/L) | 18.17±5.20 | 18.17±5.71 | 18.54±4.81 | 0.095 | 0.909 |
| Scr (μmol/L) | 763.00(605.25,960.50) | 682.00(609.33,872.00) | 780.15(644.25,981.25) | 1.022 | 0.600 |
| PTH (pg/mL) | 344.85(228.43,483.38) | 272.50(162.98,449.93) | 272.05(169.35,358.00) | 10.238 | 0.006 |
| Sodium (mmol/L) | 142.07±3.21 | 140.95±3.52 | 141.39±3.74 | 2.123 | 0.121 |
| Potassium (mmol/L) | 4.16±0.69 | 3.99±0.76 | 4.13±0.99 | 0.725 | 0.485 |
| Calcium (mmol/L) | 2.13(2.02,2.26) | 2.16(2.01,2.21) | 2.12(1.98,2.26) | 0.482 | 0.786 |
| Phosphorus (mmol/L) | 1.41±0.38 | 1.43±0.42 | 1.46±0.33 | 0.398 | 0.672 |
| Magnesium (mmol/L) | 0.96(0.87,1.06) | 0.90(0.80,1.06) | 0.92(0.86,1.04) | 2.556 | 0.279 |
| TG (mmol/L) | 1.74(1.24,2.52) | 1.60(1.17,1.98) | 1.95(1.16,2.48) | 1.763 | 0.414 |
| TC (mmol/L) | 4.44(3.86,5.11) | 4.46(3.60,4.99) | 4.79(4.31,5.17) | 3.219 | 0.200 |
| HDL-C (mmol/L) | 1.02(0.84,1.27) | 1.17(0.89,1.31) | 1.05(0.90,1.30) | 2.138 | 0.343 |
| LDL-C (mmol/L) | 2.67(2.09,3.21) | 2.42(1.84,3.10) | 2.80(2.36,3.28) | 2.291 | 0.318 |
| Absolute handgrip strength (kg) | 56.05±16.28 | 40.41±12.89 | 41.98±12.22 | 27.620 | <0.001 |
| Relative handgrip strength | 2.54±0.77 | 1.87±0.59 | 1.88±0.67 | 26.514 | <0.001 |
| NT-proBNP (pg/mL) | 784.5(247.5,2665.75) | 756.50(293.75,1727.50) | 2315.50(590.75,4589.75) | 9.676 | 0.008 |
| LVEF (%) | 63.77±8.30 | 64.50±9.85 | 52.50±6.30 | 31.386 | <0.001 |
| LVFS (%) | 35.22±5.89 | 35.50±6.22 | 27.50±5.30 | 29.644 | <0.001 |
| LAD (mm) | 34.02±5.78 | 34.00±6.32 | 36.00±7.12 | 5.728 | 0.057 |
| LVEDD (mm) | 51.76±6.58 | 51.07±6.68 | 55.50±7.98 | 6.487 | 0.002 |
| LVESD (mm) | 32.50(29.00,37.00) | 32.50(27.75,35.25) | 36.00(32.25,43.75) | 10.549 | 0.005 |
| Urine volume (ml) | 700.00(382.00,1000.00) | 700.00(437.50,900.00) | 600.00(225.00,1012.50) | 1.262 | 0.532 |

Note: Data are presented as mean ± standard deviation for normally distributed variables, median (interquartile range) for non-normally distributed variables, and n (%) for categorical variables. P values were calculated using one-way ANOVA, Kruskal-Wallis H test, or chi-square test as appropriate. MACE, major adverse cardiovascular events; HF, heart failure; SCD, sudden cardiac death; SBP, systolic blood pressure; DBP, diastolic blood pressure; Hb, hemoglobin; CRP, C-reactive protein; FBG, fasting blood glucose; BUN, blood urea nitrogen; Scr, serum creatinine; PTH, parathyroid hormone; TG, triglycerides; TC, total cholesterol; HDL-C, high-density lipoprotein cholesterol; LDL-C, low-density lipoprotein cholesterol; LVEF, left ventricular ejection fraction; LVFS, left ventricular fractional shortening; LAD, left atrial diameter; LVEDD, left ventricular end-diastolic diameter; LVESD, left ventricular end-systolic diameter.
